# On the application of population-based structural health monitoring in aerospace engineering

**DOI:** 10.3389/frobt.2022.840058

**Published:** 2022-11-15

**Authors:** Daniel S. Brennan, Julian Gosliga, Paul Gardner, Robin S. Mills, Keith Worden

**Affiliations:** Dynamics Research Group, Department of Mechanical Engineering, University of Sheffield, Sheffield, United Kingdom

**Keywords:** population-based structural health monitoring, irreducible element modelling, transfer learning, fleet-based monitoring, aerospace engineering, data-based structural health monitoring, machine learning, knowledge transfer

## Abstract

One of the major obstacles to the widespread uptake of data-based Structural Health Monitoring so far, has been the lack of damage-state data for the (mostly high-value) structures of interest. To address this issue, a methodology for sharing data and models between structures has been developed–*Population-Based Structural Health Monitoring* (PBSHM). PBSHM works on the principle that, if populations of structures are sufficiently similar, or share sections which can be considered similar, then data and models can be shared between them for use in diagnostic inference. The PBSHM methodology therefore relies on two key components: firstly, identifying whether structures are sufficiently similar for successful transfer of diagnostics; this is achieved by the use of an abstract representation of structures. Secondly, machine learning techniques are exploited to effectively transfer information between the structures in a way that improves damage detection and classification across the whole population. Although PBSHM has been conceived to deal with large and general classes of structures, much of the detailed developments presented so far have concerned bridges; the aim of this paper is to provide similarly detailed discussions in the aerospace context. The overview here will examine data transfer between aircraft components, as well as illustrating how one might construct an abstract representation of a full aircraft.

## 1 Introduction

One of the major obstacles to the widespread uptake of Structural Health Monitoring (SHM) thus far, has been a scarcity of available damage-state data for individual structures. This lack of damage-state data arises largely because engineering structures are designed not to fail; this is particularly true of safety-critical structures like aircraft. Finding a means of “sharing” damage-state data between structures is one way of alleviating the lack of available data for a particular individual structure. One approach is so-called *Population-based Structural Health Monitoring* (PBSHM). The main objects of interest in PBSHM are *populations* or sets of structures. In the completely general case, a population might be composed of quite different structures (e.g., bridges, aircraft and wind turbines); such a population would be referred to as *heterogeneous*. At the other extreme, a population might comprise a set of nominally-identical structures (e.g., a wind-frame containing only one model of turbine); such a population would be referred to as *homogeneous*. Somewhere in between these two extremes are populations of structures grouped according to purpose; e.g., bridges *or* aircraft *or* turbines. Such populations would be heterogeneous, but would share commonalities of structure that could be exploited for PBSHM. Foundations for a general theory of PBSHM have begun with the papers: Bull et al. (2020); Gosliga et al. (2020); Gardner et al. (2020a), and an overview of the ‘story so far’ was presented in Worden et al. (2020).

Sharing data between structures can be achieved by *transfer learning*; for example, by using classifiers trained on one structure to identify damage in another [Gardner et al. (2022)], or by using a collective normal condition to detect damage in a new structure [Bull et al. (2021)]. However, when using transfer learning, one must be careful to avoid *negative transfer* [Gardner et al. (2020a)]. In a classification problem, negative transfer occurs when data are transferred in such a way that classes become mislabelled; this could have dire consequences if a classifier mistakenly identified damage-state data as healthy-state data. Therefore, care must be taken that negative transfer is avoided when transferring data between structures. In general, negative transfer is simply the situation where transfer learning makes matters *worse*.

To avoid negative transfer, there must be confidence that there is a degree of similarity between the two structures of interest. One way to avoid negative transfer would be to only transfer data between structures of the same make and model, e.g. wind turbines in a wind farm, where the major variations come from environmental conditions and manufacturing variations [Bull et al. (2020); Papatheou et al. (2015); Antoniadou et al. (2015)]. Moving beyond such homogeneous populations requires some method of assessing the *similarity* between structures, such as described in Gosliga et al. (2020); this requires some representation of the structure in question. This representation, or *Irreducible Element (IE) model* in Gosliga et al. (2020), contains information that is believed to be significant when it comes to transfer learning; it also needs to contain enough information for the problem at hand. Clearly, there need to be rules or best practice to inform the building of IE models. In the case where the populations are heterogeneous but comprise structures of only one class, there may be context-specific rules for IE models. One previous study has looked in some detail about the IE modelling of bridges [Gosliga et al. (2022)]; one of the major objectives of this current paper is to look at the case of aerospace structures in similar detail.

IE models can also be useful when CAD models or technical drawings are not available, since they can provide a coarse and non-parameterised representation of a given structure, they can provide additional information to inform and improve transfer learning. IE models can inform transfer learning by showing that the structures are correlated in some physical sense as opposed to only sharing correlations in the data. For example, IE models can also provide confidence that the causal mechanisms behind damage are the same. IE models can also potentially be shared without revealing commercially-sensitive information.

The aim of the current paper is to introduce the concepts of PBSHM, specifically in the aerospace context. This breaks down into two main problems; in the first case, it has been observed above that some means is necessary for comparing the similarity of two structures, namely IE models and their attributed graphs. This paper will discuss some of the details of building IE models specifically for aerospace structures and components and illustrate the process *via* a full aircraft case study. In the second place, the paper will demonstrate how this comparison of structural similarity can be used to inform knowledge transfer between aerospace structures—specifically here for two aircraft wings. This knowledge transfer involves the key machine learning tool in PBSHM–transfer learning. The paper also discusses how the IE model generated for the full aircraft structure differs from the models used to describe the two wings, and why these differences exist.

To achieve these aims, the layout of the paper is as follows: Section 2 briefly introduces two key concepts within PBSHM. The first of these two key concepts is a method called *Irreducible Element* (IE) modelling, which is used to produce an abstract representation of a structure which is relevant to the particular SHM problem. The second of these key PBSHM concepts is that of *attributed graphs*, which allow IE models to be compared *via* graph-matching. Section 3 features a case study which highlights a third key concept within PBSHM–transfer learning–by describing how knowledge can be transferred between two aircraft substructures, namely a wing from a Folland Gnat aircraft to a wing from a Piper Tomahawk. Section 4 then describes the process for generating an IE model for a full physical structure, in this case an RAF Hawk T. Mk1. Section 5 discusses the differences between the IE models generated for the Gnat and Piper wings, and the Hawk.

## 2 Population-based structural health monitoring

The core steps for achieving PBSHM within heterogenous populations of structures are: generating the IE model along with the attributed graph of the structure, comparing the graphs for two different structures, and then performing some form of transfer learning [Gardner et al. (2020a)]. This section will describe these three aspects of PBSHM.

### 2.1 Irreducible element models

As mentioned previously, IE models provide a description of a structure that allows one to determine whether it is similar to another structure. This description needs to be detailed enough for the problem at hand, while not containing so much information that similarities are either obscured, or comparison becomes inefficient. The IE model also needs to contain information that is important to avoid negative transfer for a given problem; such information might be specific to the class of structures concerned. A detailed description of the general theory of IE models can be found in Gosliga et al. (2020); as an example of how rules for IE-model generation arise for specific classes of structures, the reader can consult Gosliga et al. (2022), which is focussed on bridges. It should be noted that since both these papers have been published, the theory and terminology and have been updated and standardised; for the most up-to-date version, the reader is referred to Brennan et al. (2021). Part of this paper Section 4 and Section 5 will be concerned with IE models for aerospace structures.

First of all, when generating an IE model, the structure must be subdivided into elements that correspond to the resolution of the SHM problem being considered. For example, if the SHM problem at hand was largely concerned with features governed by the overall flight dynamics of an aircraft, it would not be worth breaking the structure down into individual rivets and bolts; a better approach may be to treat the aerodynamic surfaces as single elements, while keeping control surfaces and the fuselage separate. (Although, as will be seen in Section 4, to accurately describe the geometry of aerospace components, further subdivisions may be necessary).

Each of these elements then requires attributes. Attributes describe the properties and classification for aspects of the elements believed to have relevance to transfer learning [Gardner et al. (2020a)]. These attributes are: both the material properties, and the type of material (e.g., metals, polymers, composite); and the type of geometry of the element (e.g., plate, beam, shell), along with its dimensions.

A further set of attributes that can be applied to a given element are *contextual labels*, which describe something about the function of the element, for example if the element represents a wing or a fuselage. By describing the function of an element, the contextual label may also provide some additional information about the loads that the said element may experience. For example, a beam and a column may possess the same geometry, and yet the loading they experience is different. Contextual labels can also provide a useful method for differentiating between different types of structure; for example, consider the case where an IE model contains an element with the contextual label “wing,” it would be fair to say that this is likely to be the IE model for an aircraft, and conversely, unlikely to represent a bridge. While it may not be optimal to consider populations so heterogeneous that they contain completely different classes of structures, the general theory of PBSHM should be able to accomodate the situation. The other reason why completely general populations may be of interest is because quite different structures may yet contain similar *substructures*.

A description of the layout, or topology, of any structure is important for ensuring that two structures are similar enough that data transfer is possible. The overall topology of the structure is captured in the relationships between elements. In aircraft, the topology of the structure is analogous to configuration of the aircraft (location and number of engines, tailplane arrangement, etc.), and differences in the configuration have a clear effect on the overall flight dynamics of a given aircraft, as well as implications for damage localisation. It is also important to note that structural similarity is not *sufficient* for positive transfer, one also should take care that features measured on the structures can be compared meaningfully. At the risk of stating the obvious, it would be unlikely that transient acoustic emission waves measured on one structure could be effectively transferred onto frequency response functions measured on another.

Constitutive materials affect the dynamics of the structure, and also the failure modes that exist. There are clear differences in the failures of composite components (delamination) that do not exist in aluminium components–and vice versa. These differences in the type of failures that can occur for a specific type of material mean that there will be damage labels that cannot be transferred. However, there will be some cases where these differences matter less, such as when examining the overall dynamics. However, when examining the overall dynamics of the structure, the actual properties of the material will play more of a role, and so these need to be included as well.

The geometry again will have some effect on the overall dynamic behaviour, given the differences in how beams and plates behave, for example. There are also localisation issues if certain components are similar shapes but some have holes or areas that do not correspond between them. The size of components within a structure also influences the dynamics of the structure and so this will affect whether knowledge transfer is possible.

It can be seen that, for each of these categories, and more generally, there will be SHM problems for which certain differences are more important, and others are less.

Section 4 here goes into the details of generating an IE model for a particular aircraft (Hawk T. Mk1); a sample of the elements for the fuselage section of the Hawk can be found in Table 1. In this table, the name of each element is included, along with a description of its material type and its geometrical type. In this table, arrows represent the sub-categories used to describe either the geometric or material type of an element, for example, the antenna is made of an aluminium alloy, which is a type of metal. The geometrical sub-categories are a little less intuitive as they are specific to the IE model schema [Brennan et al. (2021)] that has been designed.

**TABLE 1:** Regular Elements for the Fuselage and Vertical Stabiliser of the generalised Hawk T. Mk1. As mentioned in the text, arrows point from less specific to more specific descriptors. For example, *aluminium alloy* is more specific than *metal*. The term “translate and scale” refers to a type of element which does not change the shape of its cross section along a length dimension, but the shape may grow or shrink (scale). A circular cone would of this type, and a circular cylinder would be specific type of cone.

Element Name	Contextual Type	Material Type	Geometry Type
Fuselage			
antenna	Other	Metal → Aluminium Alloy	Solid → Translate and Scale → Cylinder
fuselage-a	Fuselage	Metal → Aluminium Alloy	Shell → Translate and Scale → Cylinder
fuselage-b	Fuselage	Metal → Aluminium Alloy	Shell → Translate and Scale → Cylinder
fuselage-c	Fuselage	Metal → Aluminium Alloy	Shell → Translate and Scale → Cylinder
fuselage-d	Fuselage	Metal → Aluminium Alloy	Shell → Translate and Scale → Cylinder
fuselage-e	Fuselage	Metal → Aluminium Alloy	Shell → Translate and Scale → Cylinder
fuselage-f	Fuselage	Metal → Aluminium Alloy	Shell → Translate and Scale → Cylinder
fuselage-g	Fuselage	Metal → Aluminium Alloy	Shell → Translate and Scale → Cylinder
fuselage-h	Fuselage	Metal → Aluminium Alloy	Shell → Translate and Scale → Cylinder
fuselage-i	Fuselage	Metal → Aluminium Alloy	Shell → Translate and Scale → Cylinder
fuselage-j	Fuselage	Metal → Aluminium Alloy	Shell → Translate and Scale → Cylinder
fuselage-k	Fuselage	Metal → Aluminium Alloy	Shell → Translate and Scale → Cylinder
fuselage-l	Fuselage	Metal → Aluminium Alloy	Shell → Translate and Scale → Cylinder
Vertical Stabiliser			
vertical-stabiliser-a	Aerofoil	Metal → Aluminium Alloy	Shell → Translate and Scale → Cylinder
vertical-stabiliser-b	Aerofoil	Metal → Aluminium Alloy	Shell → Translate and Scale → Cylinder
vertical-stabiliser-c	Aerofoil	Metal → Aluminium Alloy	Shell → Translate and Scale → Cylinder
vertical-stabiliser-d	Other	Metal → Aluminium Alloy	Shell → Translate and Scale → Cylinder
rudder	Other	Metal → Aluminium Alloy	Shell → Translate and Scale → Cylinder

#### 2.1.1 Schema

The schema defines the data structure for the IE models, as well as determining which values are allowed in which fields. For example, in Table 1, the schema has a list of geometry types that are allowable. The schema also requires that the data be structured according to the sub-categories defined within the schema. In this case, it would never allow the user to simply enter “Cylinder,” the user must provide extra details to tell whether it is a solid or shell, as well as how the shape is to be defined.

The schema is crucial in ensuring that IE models provided to the database are all constructed to the same standards and use the same set of rules for specific classes of structure. It is important that IE models are standardised to ensure that any differences picked up during the similarity comparison step are a result of differences in the structure itself and not differences in how the user has described the model. As work progresses, it is hoped that the similarity comparison stage can be made more robust, but currently it is far easier to enforce rules and conventions for generating IE models. However, rules and conventions for generating IE models will always remain important for ensuring consistency in how the framework is applied, which improves confidence in the results provided.

As the schema only allows the user to enter information that is certain to be consistent with the data that already exists within the database, it must be extended when new types are encountered. For example, if one designed the schema around aeroplanes only, the material type Cement would not be included. Therefore, types such as “Other” are included, which indicate that for a new type of structure, additional types may be required. The work described in Brennan et al. (2022) is an example of where, *via* the process of trying to describe an aircraft structure, new types are discovered, which will then be incorporated into the schema.

#### 2.1.2 Relationships

Once the elements of an IE model have been defined, it is then necessary to define the relationships between them. These relationships can either be physical, referred to here as *joints*, or abstract, of which there are several varieties. Further descriptions of the types of relationships can be found in Brennan et al. (2021).

The joints within a structure are intuitive, representing the physical relationship between two elements. For example, two elements may be welded together. The properties of this joint can be described; it exists in reality. Welds are an example of a *static* joint. The other type of joint that exists is a *dynamic* joint, where there is at least one degree of freedom in which the two elements may move relative to one another. This could be a ball-and-socket joint, or a hinge joint, or some form of slider.

The other types of relationships are more abstract. One type of abstract relationship is a *perfect* relationship, in which a larger part is split into smaller elements, but these elements do not correspond to separate structural components. The divisions are instead based on the SHM problem; for example, dividing the Gnat and Piper wings into sections to aid with damage localisation, as described in Section 3 and Gardner et al. (2022). Another example of this is shown in Figure 6, where the wing is subdivided into smaller elements in order to more accurately capture the geometry.

The other type of abstract relationship is the *boundary* relationship, which simply denotes where the elements that make up a structure attach to the ground elements (described in Section 2.1.3). These boundary relationships mark the end of the description of the structure, as anything beyond these boundary relationships must be a ground element, and as such contains no further structural information. As a simple example, if one is only concerned with the wing of an aircraft, the connection to the fuselage could be considered as a connection to ground. In contrast, if the entire structure is of interest, the ground elements would be connected to the bases of the landing gear and would represent literal ground.

Examples of the types of relationships that can be found within a structure are shown in Table 2, which denotes the relationships within the fuselage section of the aircraft in question. This table shows both static and dynamic joints, as well as the perfect joints between elements in the wing.

**TABLE 2:** Relationships for the Fuselage and Vertical Stabiliser of the generalised Hawk T. Mk1.

Relationship Name	Element Set	Type
Fuselage		
antenna-fuselage-a	{antenna, fuselage-a}	Joint → Static
fuselage-a-b	{fuselage-a, fuselage-b}	Perfect
fuselage-b-c	{fuselage-b, fuselage-c}	Perfect
fuselage-c-d	{fuselage-c, fuselage-d}	Perfect
fuselage-d-e	{fuselage-d, fuselage-e}	Perfect
fuselage-e-f	{fuselage-e, fuselage-f}	Perfect
fuselage-f-g	{fuselage-f, fuselage-g}	Perfect
fuselage-g-h	{fuselage-g, fuselage-h}	Perfect
fuselage-h-i	{fuselage-h, fuselage-i}	Perfect
fuselage-i-j	{fuselage-i, fuselage-j}	Perfect
fuselage-j-k	{fuselage-j, fuselage-j}	Perfect
fuselage-k-l	{fuselage-k, fuselage-l}	Perfect
Fuselage to Vertical Stabiliser		
fuselage-h-vertical-stabiliser-a	{fuselage-h, vertical-stabiliser-a}	Joint → Static
fuselage-i-vertical-stabiliser-b	{fuselage-i, vertical-stabiliser-b}	Joint → Static
fuselage-j-vertical-stabiliser-b	{fuselage-j, vertical-stabiliser-b}	Joint → Static
fuselage-j-vertical-stabiliser-d	{fuselage-j, vertical-stabiliser-d}	Joint → Static
fuselage-k-vertical-stabiliser-d	{fuselage-k, vertical-stabiliser-d}	Joint → Static
fuselage-l-vertical-stabiliser-d	{fuselage-k, vertical-stabiliser-d}	Joint → Static
Vertical Stabiliser		
vertical-stabiliser-a-b	{vertical-stabiliser-a, vertical-stabiliser-b}	Perfect
vertical-stabiliser-b-c	{vertical-stabiliser-b, vertical-stabiliser-c}	Perfect
vertical-stabiliser-b-d	{vertical-stabiliser-b, vertical-stabiliser-d}	Joint → Static
vertical-stabiliser-b-rudder	{vertical-stabiliser-b, rudder}	Joint → Dynamic

#### 2.1.3 Ground elements

Ground elements (as shown in Table 3), represent bodies to which the structure that is being modelled is attached, but are themselves not being modelled. In the case of the aircraft described here, this is literally the ground. In some other cases this may be a strong wall, or if say, one wished to only model the wing of an aeroplane, then the fuselage it was attached to could be treated as ground.

**TABLE 3:** Ground Elements for the Landing Gear of the generalised Hawk T. Mk1.

Element Name	Type
Landing gear	
right-ground	Ground
left-ground	Ground
center-ground	Ground

### 2.2 Attributed graphs

Once the structure has been subdivided into elements, and the attributes (geometrical, material, contextual etc.), for each element have been decided upon, this information can be extracted in the form of an attributed graph. IE models are currently created in spreadsheets and other human-friendly formats, while the attributed graph extracts the topological information, making it easier to process using graph-matching algorithms or other graph comparison techniques.

The information contained within the IE model and the attributed graph is identical and the two objects are in direct correspondence; however, the attributed graph explicitly expresses the topology in the form of a neighbourhood list for each element, whereas in the IE model, the topology is stored in the element sets of the relationships. Using the element sets of each relationship to define the topology is identical to defining a graph by its vertex set and edge set. Nonetheless, the distinction between the IE model and the attributed graph remains useful for descriptive purposes, as people associate the IE model with some form of physical model that can be visualised, and associate the attributed graph with some more abstract form of the same data.

Treating the information contained within the IE model as a graph, allows a whole suite of graph-matching tools to be used to determine the similarity between two structures. one group of such tools contains exact graph-matching algorithms which search for the common substructures between two structures [Bron and Kerbosch (1973); Koch (2001); Cao et al. (2008); van Berlo et al. (2013); Raymond et al. (2002)]. For an in-depth review of maximum common subgraph (MCS)-based approaches with a focus on biology and chemistry, the reader is referred to Duesbury et al. (2017). Others are based on deep learning using neural networks [Li et al. (2019); Hamaguchi et al. (2017); Beck et al. (2018); Sutskever et al. (2014); Duvenaud et al. (2015); Liao et al. (2019)]; a more thorough review on graph matching using neural networks can be found in Bacciu et al. (2020).

## 3 Gnat and Piper Tomahawk aircraft wing case study

The abstract respresentations of structures described in the previous section form part of a wider methdology for performing PBSHM [Bull et al. (2020); Gosliga et al. (2020); Gardner et al. (2020a); Tsialiamanis et al. (2021)]. As mentioned in the introduction, the key components of this methodology are: 1) determining how structurally similar structures in a population are, using AGs and graph matching algorithms [Gosliga et al. (2020), and distance metrics (Wickramarachchi et al. (2021)], and 2) pooling knowledge from the datasets that correspond to these structures [Bull et al. (2020); Gardner et al. (2020a); Tsialiamanis et al. (2021); Gardner et al. (2020b); Bull et al. (2021)]—typically performed using transfer learning [Gardner et al. (2020a)]. This section presents a case study demonstrating how abstract representations of structures are used in transferring knowledge between members of a population.

The population are two aircraft wings, one from a Gnat trainer aircraft [Worden et al. (2003); Manson et al. (2003a,b)], and the other a Piper Tomahawk aircraft [Barthorpe et al. (2017)], forming a heterogenous population. The SHM scenario is to perform damage localisation on the Piper Tomahawk wing using labelled damage observations on the Gnat wing (i.e. the Piper Tomahawk dataset is unlabelled). Damage in this case study is simulated by the removal of inspection panels, as it was not possible to damage the wings [Worden et al. (2003); Manson et al. (2003a,b); Barthorpe et al. (2017)]. The Gnat aircraft wing has nine inspection panels and the Piper Tomahawk has five inspection panels. The PBSHM challenge is to transfer damage localisation knowledge from the Gnat to the Piper Tomahawk, which allows a classifier trained on the Gnat dataset to be used in localising damage on the Piper Tomahawk. The PBSHM methodology must therefore select which combination of localisation labels from the Gnat, out of a possible 15120 combinations, gives the best performance, when paired with corresponding localisation labels from the Piper. This number of combinations comes from the fact that the problem is “9-choose-5” where the permutation order also matters (5 out of 9 labels on the Gnat can be paired with the 5 from the Piper), meaning 126 × 120 = 15,120.

The Gnat and Piper Tomahawk datasets both consist of transmissibility features–the magnitude of the transmissibility between 1,024 and −2048 Hz in 1 Hz frequency bins–with a set of two uniaxial accelerometers forming a transmissibility path that targets a particular inspection panel of interest [for more details see Worden et al. (2003); Manson et al. (2003a,b); Barthorpe et al. (2017)].

### 3.1 Identifying structural similarities

**FIGURE 1:**
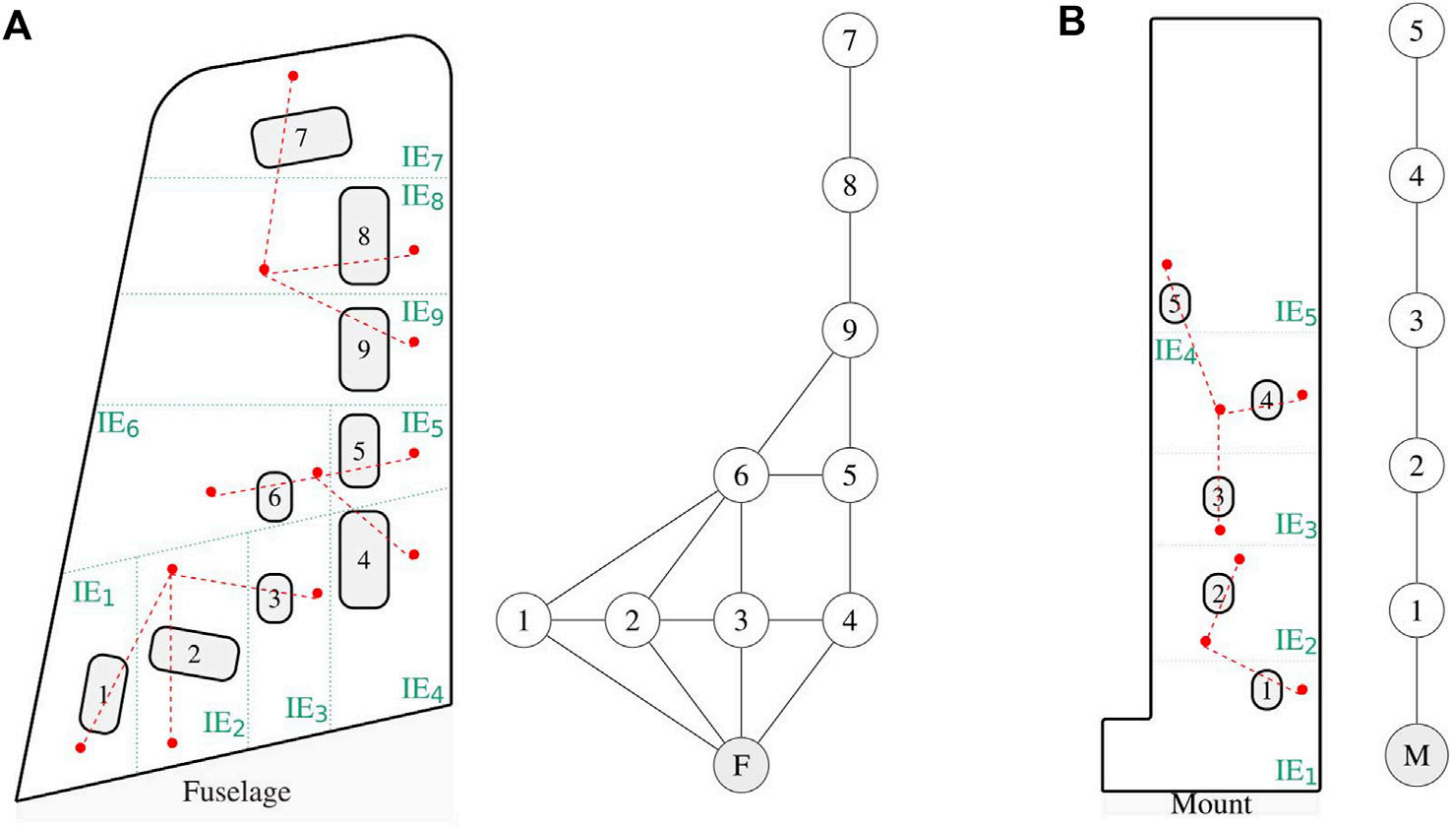
Schematic **(A)** of the Gnat aircraft wing (not to scale) and associated irreducible element model (left) with its corresponding attributed graph (right). Schematic **(B)** of the Piper Tomahawk aircraft wing (not to scale) and associated irreducible element model (left) with its corresponding attributed graph (right).

As proposed in this paper, structural similarities can be objectively quantified by the use of graphical representations of structures and the use of metrics in the graphical domain. The process involves developing an IE model, converting that into an attributed graph and performing graph matching with representations of other structures. The resolution or granularity of an IE model depends on the PBSHM task. For instance, in this case study the IE model for each wing is required to capture the topology with respect to the inspection panel locations. The IE models, and their corresponding AGs, for the Gnat and Piper Tomahawk wings are depicted in Figure 1. The relationships between each IE are modelled as ‘perfect’ connections, as further resolution about rivet and stiffener locations are not required for obtaining the key differences between the inspection panel locations. The IE models are mapped onto AGs where the node numbering has been kept consistent with the inspection panel numbering.

The AGs in Figure 1 can be compared using a graph-matching algorithm. Here, the modified Bron-Kerbosch (BK) algorithm is utilised as in Gosliga et al. (2020), identifying four maximum common subgraphs (MCSs) shown in Table 4. The set of MCSs is a significant 99.97% reduction on the 15120 potential combinations of Gnat localisation labels that could be mapped onto the Piper Tomahawk. Further engineering insight would also suggest that MCS 1) is the most likely to produce successful transfer, as the set of nodes in the Gnat and Piper Tomahawk AGs are both ‘cantilever-like’ on the trailing edge of the wings.

**TABLE 4:** Maximum common subgraphs between the Gnat and Piper Tomahawk attributed graphs using the modified BK algorithm.

Piper		M	1	2	3	4	5
Gnat	(a)	F	4	5	9	8	7
	(b)	F	1	6	9	8	7
	(c)	F	2	6	9	8	7
	(d)	F	3	6	9	8	7

### 3.2 Transferring knowledge

Transfer learning is an important part of PBSHM, allowing labelled information from *source* datasets to diagnose unlabelled *target* datasets [Gardner et al. (2020b); Gardner et al., (2021)]; Bull et al. (2021)]. This process allows PBSHM to overcome issues that hinder conventional data-driven approaches to SHM, such as a lack of available health-state data. The papers Gardner et al. (2020b, 2021), utilise domain adaptation [Pan and Yang (2010); Weiss et al. (2017); Zhuang et al. (2021)], a branch of transfer learning, that seeks to harmonise the (labelled) source and (unlabelled) target datasets in a latent space, such that a classifier trained on the source dataset in the latent space generalises to the target dataset.

Domain adaptation is performed on the four MCSs in Table 4 using balanced distribution adaptation (BDA) [Wang et al. (2017)]. The algorithm minimises an approximation of the distance between the source $\left(D_{s}=\left\{X_{s},{\vec{y}}_{s}\right\}\right)$ and target ( $D_{t}=\left\{X_{t},{\hat{\vec{y}}}_{t}\right\}$ , where ${\hat{\vec{y}}}_{t}$ are psuedo-labels) joint distributions,

$$D\left(D_{s},D_{t}\right)\approx \left(1-\lambda \right)D\left(P\left(X_{s}\right),P\left(X_{t}\right)\right)+\lambda D\left(\frac{P\left({\vec{y}}_{s}\right)P\left(X_{s}\;|\;{\vec{y}}_{s}\right)}{P\left(X_{s}\right)},\frac{P\left({\hat{\vec{y}}}_{t}\right)P\left(X_{t}\;|\;{\hat{\vec{y}}}_{t}\right)}{P\left(X_{t}\right)}\right)$$ (1)

achieved *via* a weighted maximum mean discrepancy (MMD) cost function [i.e., *D* is the maximum mean discrepancy distance Wang et al. (2017)], where *λ* ∈ [0, 1] is the weight that “balances” the contribution of the marginal or conditional distributions. In this paper *λ* = 1, meaning that BDA assumes that the marginals are aligned and that the class conditionals are the cause of domain shift. By utilising the low-rank empirical kernel embedding $\tilde{K}=KAA^{T}K$ [Schölkopf et al. (1998)], the MMD cost function can be minimised using a Lagrangian approach in order to identify a linear mapping *A* on a kernel matrix *K* = *k* (*X*, *X*′) where *X* = *X*_*s*_ ∪ *X*_*t*_. The inferred latent space is therefore $Z=KA\in R^{\left(N_{s}+N_{t}\right)\times k}$ , where *k* is the dimension of the latent space (*k* < *d*). For the complete details of BDA the author is referred to Wang et al. (2017).

**FIGURE 2:**
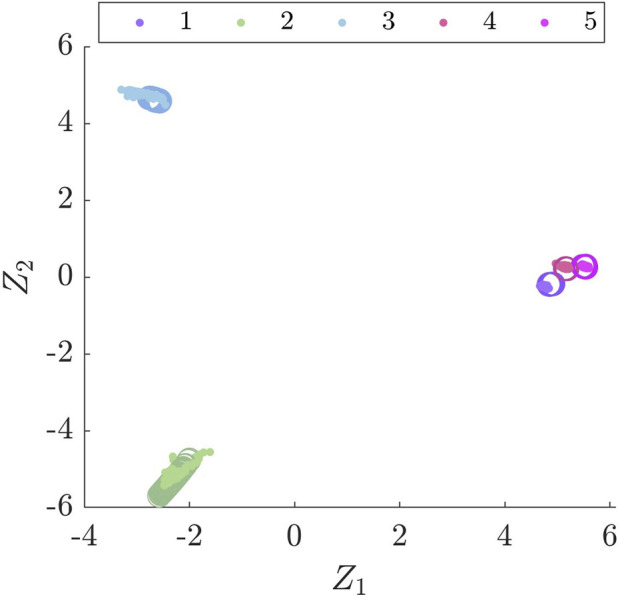
A visualisation of the BDA latent space for candidate set (a), where • denotes the Gnat and ○ denotes the Piper Tomahawk datasets.

A *k*-nearest neighbours (kNN) classifier is trained on the Gnat (source) dataset in the latent space identified by BDA and used to classify the Piper Tomahawk (target) dataset. F1 scores are presented in Table 5 for the four MCSs, where the F1 score is a commonly-used measure of classification accuracy and is defined as the harmonic mean of the recall and precision metrics. It can be seen that MCS 1) produces 100% classification accuracy on the Piper Tomahawk, showing that it is possible to transfer knowledge from the Gnat to the Piper Tomahawk aircraft without performance loss. A visualisation of the transfer component space for MCS 1) is shown in Figure 2.

**TABLE 5:** F1 (comparison) scores for the four maximum common subgraphs.

Subgraph set		(a)	(b)	(c)	(d)
F1	Gnat	1.0	1.0	1.0	1.0
	Piper Tomahawk	1.0	0.4	0.2	0.6

In order to understand the usefulness of the abstract representation of structures, transfer learning and classification was performed for all 15120 combinations. It was found that only 92 combinations produced F1 scores of 1 on the Piper Tomahawk, which is around 0.6% of the total. This result shows that leveraging structural information reduces the computational burden required to perform PBSHM. The process of building IE models, AGs and performing graph-matching has led to a probability of the perfect F1 score being 0.25 instead of 0.006, demonstrating the effectiveness of the proposed methodology. This process has also yielded an association of labels that matches engineering intuition, by aligning panels that corresponding to “cantilever-like” sections in each wing.

## 4 Irreducible element model for an aeroplane

The previous section has demonstrated how IE models can be used to inform transfer learning for aircraft components. This section describes how IE models can be constructed to describe a full aircraft–a Hawk T. Mk1.

**FIGURE 3:**
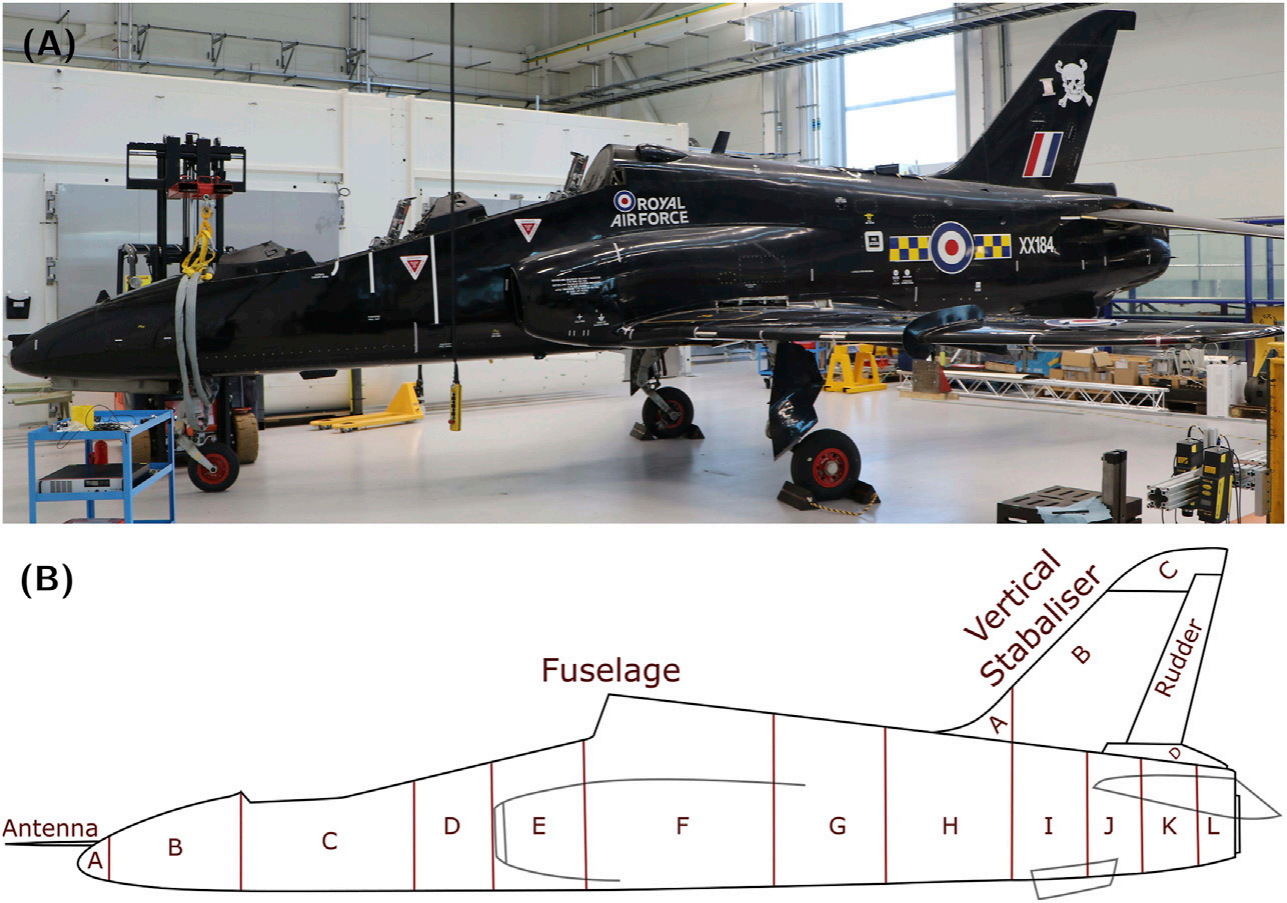
**(A)** Photo of the side view of the Hawk T. Mk1 at the Laboratory for Verification and Validation. **(B)** Irreducible Element model breakdown of the center section of the Hawk T. Mk1.

The Hawk T. Mk1 is the end result of an RAF project to replace the aircraft in their fast jet pilot training programme. A detailed history of the Hawk can be found in Fraser-Mitchell (2011). The particular Hawk featured in this paper was used as the ‘bad guy’ during training exercises, hence the skull and cross bones decal visible in Figure 3A. For the purposes of this paper and the wider PBSHM work, the Hawk represents a full-size aircraft that was conveniently available for taking physical measurements, as well as other interrogations and testing.

Within an IE model it is possible to include details of the structure down to the nuts and bolts; however, in most cases this would not be required, and an IE model would only be generated to the level of detail required for the intended use case. Brennan et al. (2022) describe the process of generating an IE model of the Hawk T. Mk1 at the *Laboratory for Verification and Validation* (LVV) in Sheffield, United Kingdom. For the IE model described, the stated aim was to generate a model that described the Hawk’s overall airframe structure for the purpose of determining the similarities between itself and other aeroplanes. Being able to include more detail at a later date opens up the possibility for future revisions of the IE model of the Hawk, where a more detailed look into specific systems/the whole structure may be desired for damage localisation, such as individual panels or sub systems like the avionic systems.

Because the IE model in question is only of the Hawk’s airframe, the level of detail required is one that captures the overall geometry of the Hawk aircraft. Comparing the overall geometry of various aircraft may be useful when looking for aircraft with similar configurations in terms of engines, wings, control surfaces, etc. Such similar configurations should have some similarity in terms of flight dynamics. To capture the overall geometry of the Hawk, the elements chosen subdivide the major components of the aircraft into smaller elements to facilitate improved representation of its geometry. For example, to accurately capture their geometry, the wings are subdivided into multiple elements along their length. This is necessary as the cross-section of each wing varies considerably along its length. Another area where it was necessary to subdivide one of the major components of the aeroplane was the fuselage of the Hawk, as the air intakes and other features of the aircraft create a complex geometry.

**FIGURE 4:**
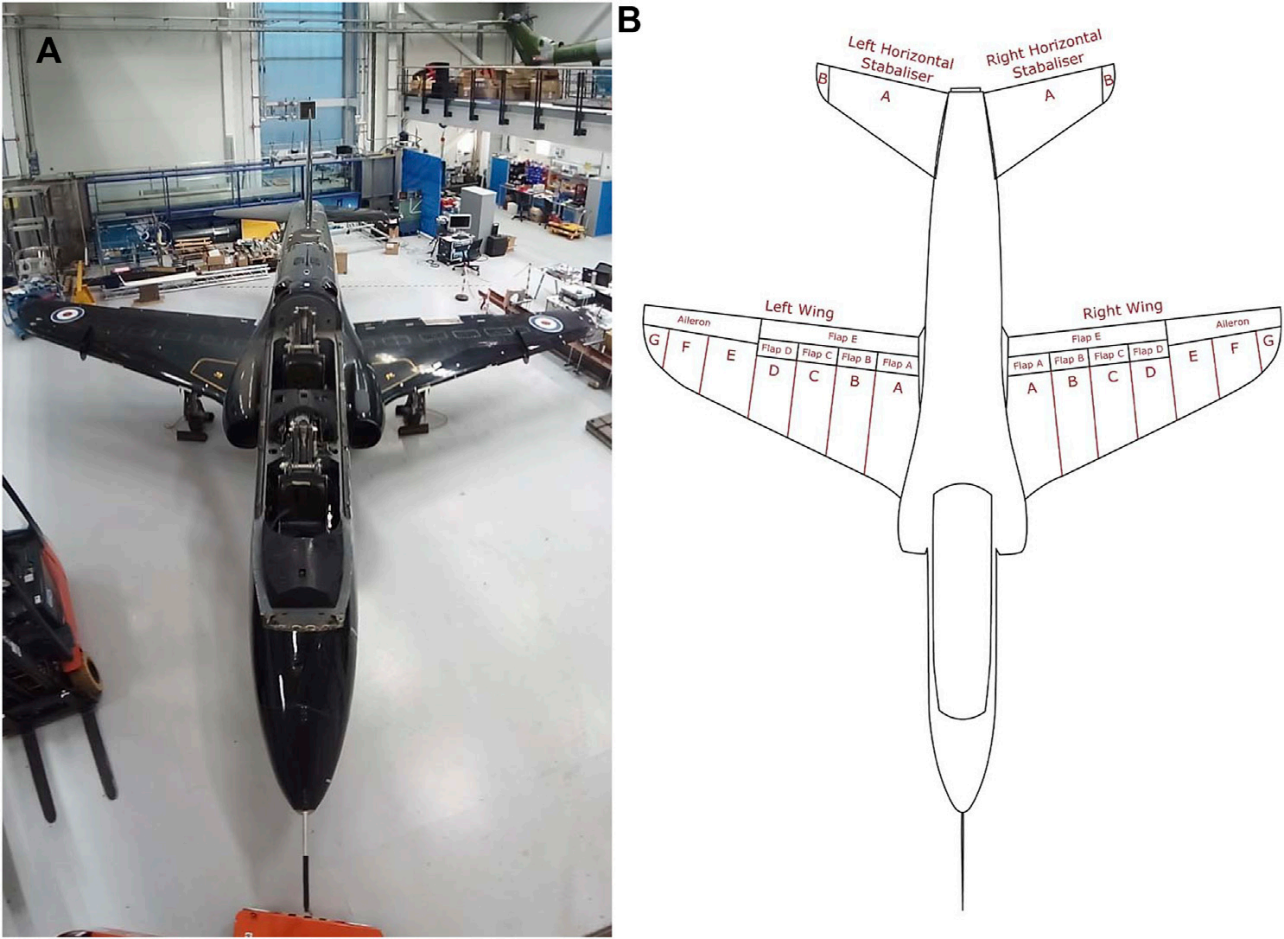
**(A)** Photo of the top down view of the Hawk T. Mk1 at the Laboratory for Verification and Validation. **(B)** Irreducible Element model breakdown of the wing and horizontal stabiliser sections of the Hawk T. Mk1.

**FIGURE 5:**
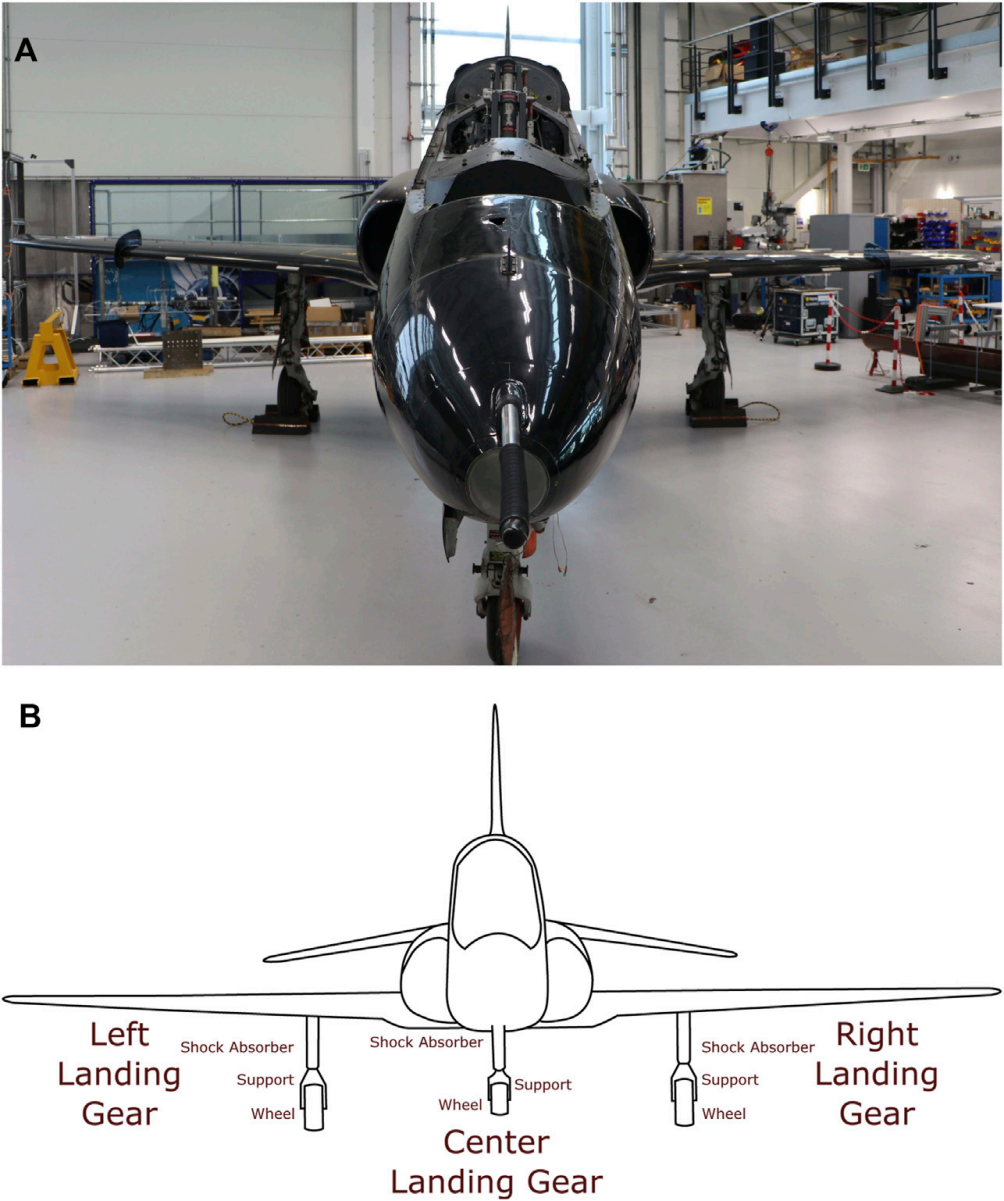
**(A)** Photo of the front view of the Hawk T. Mk1 at the Laboratory for Verification and Validation. **(B)** Irreducible Element model breakdown of the landing gear of the Hawk T. Mk1.

Given the approach described above, the aircraft was divided into three major sections, namely: the body (see Figure 3A), which consists of the fuselage and vertical stabiliser; the aerofoils (see Figure 4A), which consists of both wings and both horizontal stabilisers; and finally, the landing gear (see Figure 5A), which consists of the left, center and right landing gear. This approach of dividing a structure into major components is similar to the process using in Gosliga et al. (2022) for IE model generation from bridges, where each bridge was divided into sections corresponding to the deck, the supports, the foundations, etc.

Each sub-component within a section required a different approach for dividing said sub-component into elements. Figure 3 depict how the fuselage and vertical stabilisers were divided into their corresponding elements. The division of the fuselage matches construction markers on the surface of the body; i.e. where there is a visible delimiter *via* either rivets or welds holding together multiple pieces of sheet metal together. However, the vertical stabilisers have been divided according to curvature changes along the leading edge. The elements and relationships for the body section are listed in Tables 1, 2, respectively.

In contrast, within the aerofoil section, the wings (depicted in Figure 4) have been divided inline with the control surfaces at the rear of the wing. The horizontal stabilisers, however, are divided into elements according to changes in curvature along the leading edge, similar to the vertical stabilisers. Tables 6, 7 list the elements and relationships for the aerofoils.

**TABLE 6:** Regular Elements for the Right Wing, Horizontal Stabiliser Right, and Landing Gear of the generalised Hawk T.Mk1. See Section 6.1.1 and Section 6.2.1 for the Left and Center counterparts of the generalised Hawk T. Mk1.

Element Name	Contextual Type	Material Type	Geometry Type
Right wing			
right-wing-a	Wing	Metal → Aluminium Alloy	Shell → Translate and Scale → Cylinder
right-wing-b	Wing	Metal → Aluminium Alloy	Shell → Translate and Scale → Cylinder
right-wing-c	Wing	Metal → Aluminium Alloy	Shell → Translate and Scale → Cylinder
right-wing-d	Wing	Metal → Aluminium Alloy	Shell → Translate and Scale → Cylinder
right-wing-e	Wing	Metal → Aluminium Alloy	Shell → Translate and Scale → Cylinder
right-wing-f	Wing	Metal → Aluminium Alloy	Shell → Translate and Scale → Cylinder
right-wing-g	Wing	Metal → Aluminium Alloy	Shell → Translate and Scale → Cylinder
right-flap-a	Aerofoil	Metal → Aluminium Alloy	Shell → Translate and Scale → Cylinder
right-flap-b	Aerofoil	Metal → Aluminium Alloy	Shell → Translate and Scale → Cylinder
right-flap-c	Aerofoil	Metal → Aluminium Alloy	Shell → Translate and Scale → Cylinder
right-flap-d	Aerofoil	Metal → Aluminium Alloy	Shell → Translate and Scale → Cylinder
right-flap-e	Aerofoil	Metal → Aluminium Alloy	Shell → Translate and Scale → Cylinder
right-aileron	Aerofoil	Metal → Aluminium Alloy	Shell → Translate and Scale → Cylinder
Right Horizontal Stabiliser			
right-horizontal-stabiliser-a	Aerofoil	Metal → Aluminium Alloy	Shell → Translate and Scale → Cylinder
right-horizontal-stabiliser-b	Aerofoil	Metal → Aluminium Alloy	Shell → Translate and Scale → Cylinder
Right Landing Gear			
right-shock-absorber	Other	Metal → Aluminium Alloy	Solid → Translate → Cylinder
right-support	Other	Metal → Aluminium Alloy	Plate → Other
right-wheel	Wheel	Other	Other

**TABLE 7:** Relationships for the Right Wing, Horizontal Stabiliser, and Right Landing Gear of the generalised Hawk T.Mk1. See Section 6.1.2 and Section 6.2.2 for the Left and Center counterparts.

Relationship Name	Element Set	Type
Right wing		
right-wing-a-b	{right-wing-a, right-wing-b}	Perfect
right-wing-b-c	{right-wing-b, right-wing-c}	Perfect
right-wing-c-d	{right-wing-c, right-wing-d}	Perfect
right-wing-d-e	{right-wing-d, right-wing-e}	Perfect
right-wing-e-f	{right-wing-e, right-wing-f}	Perfect
right-wing-f-g	{right-wing-f, right-wing-g}	Perfect
right-wing-flap-a	{right-wing-a, right-flap-a}	Joint → Dynamic
right-wing-flap-b	{right-wing-b, right-flap-b}	Joint → Dynamic
right-wing-flap-c	{right-wing-c, right-flap-c}	Joint → Dynamic
right-wing-flap-d	{right-wing-d, right-flap-d}	Joint → Dynamic
right-wing-e-aileron	{right-wing-e, right-aileron}	Joint → Dynamic
right-wing-f-aileron	{right-wing-f, right-aileron}	Joint → Dynamic
right-wing-g-aileron	{right-wing-g, right-aileron}	Joint → Dynamic
right-wing-a-flap-e	{right-wing-a, right-flap-e}	Joint → Dynamic
right-wing-b-flap-e	{right-wing-b, right-flap-e}	Joint → Dynamic
right-wing-c-flap-e	{right-wing-c, right-flap-e}	Joint → Dynamic
right-wing-d-flap-e	{right-wing-d, right-flap-e}	Joint → Dynamic
right-wing-a-fuselage-f	{right-wing-a, fuselage-f}	Joint → Static
Right Horizontal Stabiliser		
right-horizontal-stabiliser-a-b	{right-horizontal-stabiliser-a, right-horizontal-stabiliser-b}	Perfect
right-horizontal-stabiliser-a-fuselage-k	{right-horizontal-stabiliser-a, fuselage-k}	Joint → Dynamic
Right Landing Gear		
right-shock-absorber-wing-b	{right-shock-absorber, right-wing-b}	Joint → Dynamic
right-shock-absorber-support	{right-shock-absorber, right-support}	Joint → Dynamic
right-support-wheel	{right-support, right-wheel}	Joint → Dynamic
right-wheel-ground	{right-wheel, right-ground}	Boundary

Finally, the landing gear (shown in Figure 5), are divided based upon the function of each sub-component, and as such, the regular elements and relationships used for this section are also listed in Tables 6, 7. The landing gear also make contact with the ground and so feature ground elements, as listed in Table 3.

## 5 Problem-driven irreducible element models

As mentioned in Section 4, the current IE model of the Hawk has been created to capture the overall geometry of the airframe. Therefore, the current IE model of the Hawk does not include the internal components and other features of the aircraft. When creating an IE model, the information captured and the chosen division of the structure into elements is driven by the SHM context. In this case, the SHM context (or problem) is one of comparing the overall airframe with other aircraft, and so the elements have been chosen accordingly. If considering a different SHM problem, one may wish to include internal components.

Another possible SHM problem would be one based on damage localisation. This is similar to the problem posed with the Gnat and Piper wings. In this context, damage localisation was the priority, and geometry a secondary concern. This prioritisation of damage localisation is reflected in the IE models generated for both the Gnat wing (Table 8), and the Piper wing (Table 9). The geometric descriptions of elements in both the Gnat wing and the Piper wing are much simpler than that of the Hawk (Table 6). The relationships in the Gnat and Piper wing IE models are either “perfect” or “boundary,” since the IE model considers the entire wing of either aircraft to be a single component, similarly to how the wings of the Hawk have been considered, ignoring the internal construction of the wing. The internal construction of the wing was not deemed relevant to the Gnat and Piper transfer problem, with the primary emphasis on subdividing the wing, so that each panel could be identified within the IE model so that damage could be localised.

**TABLE 8:** List of elements and relationships, as well as their properties, for the Gnat aircraft wing.

Element Name	Description	Material Type	Geometry Type	Contextual Type
Gnat aircraft wing				
1	Wing Panel	Metal → Aluminium	Plate → Other	Aerofoil
2	Wing Panel	Metal → Aluminium	Plate → Other	Aerofoil
3	Wing Panel	Metal → Aluminium	Plate → Other	Aerofoil
4	Wing Panel	Metal → Aluminium	Plate → Other	Aerofoil
5	Wing Panel	Metal → Aluminium	Plate → Other	Aerofoil
6	Wing Panel	Metal → Aluminium	Plate → Other	Aerofoil
7	Wing Panel	Metal → Aluminium	Plate → Other	Aerofoil
8	Wing Panel	Metal → Aluminium	Plate → Other	Aerofoil
9	Wing Panel	Metal → Aluminium	Plate → Other	Aerofoil
Element Name	Description	Boundary	—	—
A	Fuselage	Ground	—	—
	Relationship Name	Element Set	Type	
	A	{1, 2}	Perfect	
	B	{2, 3}	Perfect	
	C	{3, 4}	Perfect	
	D	{1, 6}	Perfect	
	E	{2, 6}	Perfect	
	F	{3, 6}	Perfect	
	G	{4, 5}	Perfect	
	H	{5, 6}	Perfect	
	I	{5, 9}	Perfect	
	J	{6, 9}	Perfect	
	K	{9, 8}	Perfect	
	L	{8, 7}	Perfect	
	M	{1, A}	Boundary	
	N	{2, A}	Boundary	
	O	{3, A}	Boundary	
	P	{4, A}	Boundary	

**TABLE 9:** List of elements and relationship, as well as their properties, for the Piper Tomahawk aircraft wing.

Element Name	Description	Material Type	Geometry Type	Contextual Type
Piper Tomahawk aircraft wing				
1	Wing Panel	Metal → Aluminium	Plate → Other	Aerofoil
2	Wing Panel	Metal → Aluminium	Plate → Other	Aerofoil
3	Wing Panel	Metal → Aluminium	Plate → Other	Aerofoil
4	Wing Panel	Metal → Aluminium	Plate → Other	Aerofoil
5	Wing Panel	Metal → Aluminium	Plate → Other	Aerofoil
Element Name	Description	Boundary	—	—
A	Mount	Ground	—	—
	Relationship Name	Element set	Type	
	A	{1, 2}	Perfect	
	B	{2, 3}	Perfect	
	C	{3, 4}	Perfect	
	D	{4, 5}	Perfect	
	E	{1, A}	Boundary	

**FIGURE 6:**
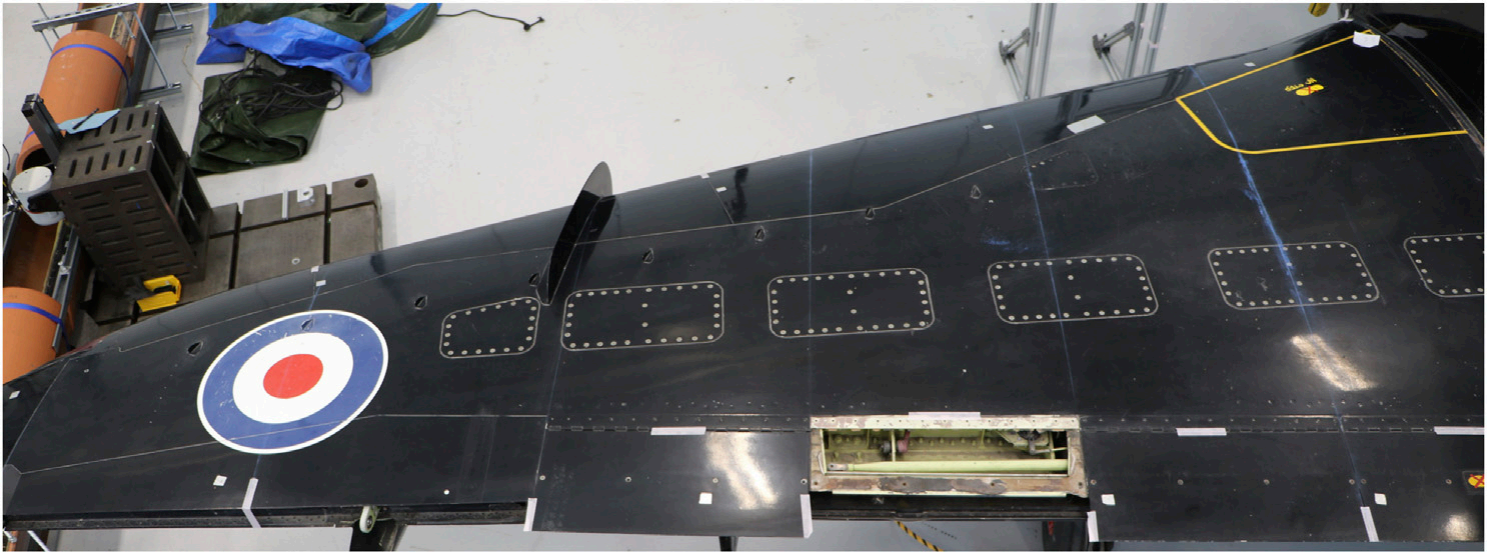
Top down view of the right wing of the Hawk T. Mk1 at the Laboratory for Verification and Validation.

The differences that focus on the subdivision of the wings can be seen by comparing the schematics of the Gnat and Piper wings (Figure 1), to the photo of the Hawk wing shown in Figure 6. From Figure 6, it can be seen that the boundaries of the elements run through several of the inspection panels of the wing, whereas in the case of the Gnat and Piper wings, the boundaries of the elements very deliberately encompass the inspection panels and avoid running through them.

If one were to pose of the problem of transferring knowledge from the Gnat wing to the Hawk wing, an alternative IE model of the Hawk wing would need to be created. An IE model of the Hawk wing which respects the inspection panel location would be required to localise damage in those areas of the wing.

## 6 Conclusion

In this paper, the PBSHM framework described in Worden et al. (2020), is specifically applied to aerospace structures. There are challenges in describing aircraft geometry that arise from its complexity, that are not found when describing the relatively simple geometries found in bridges, for example. An aircraft has many curved surfaces with aerodynamic significance, compared to some of the beam and slab constructions often found in civil infrastructure. These challenges in describing the geometry can be largely solved by careful subdivision of said geometry into appropriate elements. New geometric types will also aid in describing the geometries found in aircraft. Working out how to describe different structures, and updating the schema to accommodate these new descriptions forms a large part of the work in creating a PBSHM framework that encompasses, and remains consistent across all engineering structures.

Knowledge transfer looks promising for aerospace applications, where to date, there has been successful knowledge transfer between aircraft components following the ideas of generating IE models, examining the subgraphs between the two structures, and using this to inform which knowledge transfer will be the most successful. This is the PBSHM framework that has been laid out so far: find similarities, or level of similarity, between two or more structures and use this to inform transfer learning by selecting the most appropriate methods and associations.

The paper discusses the generation of IE models from aircraft components/substructures to a full-size jet aircraft, and through this, shows that the IE models for aircraft are very different to the IE models required for other classes of structure, with different material, geometric and even contextual labels. This is promising (and expected), in as much as it is very unlikely that bridges (for example), and aeroplanes would ever match with one another in the PBSHM database, which is the desired behaviour. In particular, there are certain contextual labels, such as ‘fuselage’, that may make it trivial to separate aircraft from bridges in the databases. This behaviour of driving structures apart (in particular bridges and aeroplanes), as more descriptive labels are included, is described in Worden et al. (2021). Furthermore, including the different material and contextual labels found in aircraft allows the PBSHM schema to be expanded, thus incorporating a greater range of engineering structures.

This paper also discusses the need for different IE models, depending on the problem at hand. For example, the current elements within the IE model of the Hawk correspond largely to changes in the overall geometry of the airframe, while the Gnat and Piper wings are divided into elements which correspond to possible damage locations. If the context for generating the IE model of a structure were to change, then a new IE model may be necessary. For example, to transfer knowledge between the Gnat and the Hawk for the purposes of localising damage within one of the wings of the Hawk, then the elements of the Hawk wing would need to be changed to respect the inspection panel location. Alternatively, if one desired a more detailed description of the Gnat or Piper wing geometry, then new IE models featuring elements that correspond to the geometry or construction would be required.

## Data Availability

The original contributions presented in the study are included in the article/Supplementary Material, further inquiries can be directed to the corresponding author.

## Appendix

**TABLE A1:** Regular elements.

Element Name	Contextual Type	Material Type	Geometry Type
Left wing			
left-wing-a	Wing	Metal → Aluminium Alloy	Shell → Translate and Scale → Cylinder
left-wing-b	Wing	Metal → Aluminium Alloy	Shell → Translate and Scale → Cylinder
left-wing-c	Wing	Metal → Aluminium Alloy	Shell → Translate and Scale → Cylinder
left-wing-d	Wing	Metal → Aluminium Alloy	Shell → Translate and Scale → Cylinder
left-wing-e	Wing	Metal → Aluminium Alloy	Shell → Translate and Scale → Cylinder
left-wing-f	Wing	Metal → Aluminium Alloy	Shell → Translate and Scale → Cylinder
left-wing-g	Wing	Metal → Aluminium Alloy	Shell → Translate and Scale → Cylinder
left-flap-a	Aerofoil	Metal → Aluminium Alloy	Shell → Translate and Scale → Cylinder
left-flap-b	Aerofoil	Metal → Aluminium Alloy	Shell → Translate and Scale → Cylinder
left-flap-c	Aerofoil	Metal → Aluminium Alloy	Shell → Translate and Scale → Cylinder
left-flap-d	Aerofoil	Metal → Aluminium Alloy	Shell → Translate and Scale → Cylinder
left-flap-e	Aerofoil	Metal → Aluminium Alloy	Shell → Translate and Scale → Cylinder
left-aileron	Aerofoil	Metal → Aluminium Alloy	Shell → Translate and Scale → Cylinder
Left horizontal stabiliser			
left-horizontal-stabiliser-a	Aerofoil	Metal → Aluminium Alloy	Shell → Translate and Scale → Cylinder
left-horizontal-stabiliser-b	Aerofoil	Metal → Aluminium Alloy	Shell → Translate and Scale → Cylinder

**TABLE A2:** Relationships.

Relationship Name	Element Set	Type
Left wing		
left-wing-a-b	{left-wing-a, left-wing-b}	Perfect
left-wing-b-c	{left-wing-b, left-wing-c}	Perfect
left-wing-c-d	{left-wing-c, left-wing-d}	Perfect
left-wing-d-e	{left-wing-d, left-wing-e}	Perfect
left-wing-e-f	{left-wing-e, left-wing-f}	Perfect
left-wing-f-g	{left-wing-f, left-wing-g}	Perfect
left-wing-flap-a	{left-wing-a, left-flap-a}	Joint → Dynamic
left-wing-flap-b	{left-wing-b, left-flap-b}	Joint → Dynamic
left-wing-flap-c	{left-wing-c, left-flap-c}	Joint → Dynamic
left-wing-flap-d	{left-wing-d, left-flap-d}	Joint → Dynamic
left-wing-e-aileron	{left-wing-e, left-aileron}	Joint → Dynamic
left-wing-f-aileron	{left-wing-f, left-aileron}	Joint → Dynamic
left-wing-g-aileron	{left-wing-g, left-aileron}	Joint → Dynamic
left-wing-a-flap-e	{left-wing-a, left-flap-e}	Joint → Dynamic
left-wing-b-flap-e	{left-wing-b, left-flap-e}	Joint → Dynamic
left-wing-c-flap-e	{left-wing-c, left-flap-e}	Joint → Dynamic
left-wing-d-flap-e	{left-wing-d, left-flap-e}	Joint → Dynamic
left-wing-a-fuselage-f	{left-wing-a, fuselage-f}	Joint → Static
Left horizontal stabiliser		
left-horizontal-stabiliser-a-b	{left-horizontal-stabiliser-a, left-horizontal-stabiliser-b}	Perfect
left-horizontal-stabiliser-a-fuselage-k	{left-horizontal-stabiliser-a, fuselage-k}	Joint → Dynamic

**TABLE A3:** Regular elements.

Element Name	Contextual Type	Material Type	Geometry Type
Center landing gear			
center-shock-absorber	Other	Metal → Aluminium Alloy	Solid → Translate → Cylinder
center-support	Other	Metal → Aluminium Alloy	Plate → Other
center-wheel	Wheel	Other	Other
Left landing gear			
left-shock-absorber	Other	Metal → Aluminium Alloy	Solid → Translate → Cylinder
left-support	Other	Metal → Aluminium Alloy	Plate → Other
left-wheel	Wheel	Other	Other

**TABLE A4:** Relationships.

Relationship Name	Element Set	Type
Left landing gear		
left-shock-absorber-wing-b	{left-shock-absorber, left-wing-b}	Joint → Dynamic
left-shock-absorber-support	{left-shock-absorber, left-support}	Joint → Dynamic
left-support-wheel	{left-support, left-wheel}	Joint → Dynamic
left-wheel-ground	{left-wheel, left-ground}	Boundary
Center landing gear		
center-shock-absorber-fuselage-b	{center-shock-absorber, fuselage-b}	Joint → Dynamic
center-shock-absorber-support	{center-shock-absorber, center-support}	Joint → Dynamic
center-support-wheel	{center-support, center-wheel}	Joint → Dynamic
center-wheel-ground	{center-wheel, center-ground}	Boundary
